# Emergence of Chikungunya Virus, Pakistan, 2016–2017

**DOI:** 10.3201/eid2602.171636

**Published:** 2020-02

**Authors:** Nazish Badar, Muhammad Salman, Jamil Ansari, Uzma Aamir, Muhammad Masroor Alam, Yasir Arshad, Nighat Mushtaq, Aamer Ikram, Javaria Qazi

**Affiliations:** National Institute of Health, Islamabad, Pakistan (N. Badar, M. Salman, J. Ansari, U. Aamir, M.M. Alam, Y. Arshad, N. Mushtaq, A. Ikram);; Quaid-I-Azam University, Islamabad (N. Badar, J. Qazi)

**Keywords:** Chikungunya virus, CHIKV, Pakistan, molecular epidemiology, vector-borne infections, arboviruses, zoonoses, viruses, *Suggested citation for this article:* Badar N, Salman M, Ansari J, Aamir U, Alam MM, Arshad Y, et al. Emergence of chikungunya virus, Pakistan, 2016–2017. Emerg Infect Dis. 2020 Feb [*date cited*]. https://doi.org/10.3201/eid2602.171636

## Abstract

During December 2016–May 2017, an outbreak of chikungunya virus infection occurred across Pakistan. The East/Central/South African genotype was predominant. This study provides baseline data on the virus strain and emphasizes the need for active surveillance and implementation of preventive interventions to contain future outbreaks.

Chikungunya is a vectorborne viral disease that causes large outbreaks, mainly in tropical and subtropical countries (*1*). The term chikungunya is derived from a word in the Makonde language (spoken in parts of Tanzania and Mozambique, Africa), kungunyala, meaning “that which bends up,” referring to the stooped posture and impaired gait patients exhibit because of severe joint pain (*2*). Chikungunya virus (CHIKV; family *Togaviridae*, genus *Alphavirus*) is an enveloped, single-strand, positive-sense RNA virus transmitted through the bite of infected *Aedes* mosquitoes, predominantly *Ae. aegypti* and *Ae. albopictus* (*2*).

West African, East/Central/South African (ECSA), and Asian CHIKV genotypes are distinguished by envelope 1 (E1) glycoprotein phylogeny. In 2005, a major outbreak in countries around the Indian Ocean was caused by the ECSA genotype (*3*). Virus mutations facilitated its replication in *Ae. albopictus* mosquitoes and its rapid spread by *Ae. albopictus* and *Ae. aegypti* mosquitoes (*3*), species prevalent in Pakistan during and after monsoon season, May–September.

In Pakistan, CHIKV was reported to be circulating in rodents as early as 1983 (*4*), but few human cases were reported. During a 2011 dengue outbreak in Lahore, some patients also had CHIKV antibodies. CHIKV emerged in Karachi during 2016, and an outbreak eventually was declared when evidence of local transmission was confirmed (*5*). We reviewed the epidemiologic and evolutionary links of CHIKV detected during December 2016–May 2017 across Pakistan.

## The Study

We tested serum samples from 584 patients with suspected CHIKV infection, according to the World Health Organization case definition (*6*). Patients were seen in different hospitals and clinics during December 20, 2016–May 31, 2017. Patients had acute onset fever (temperature >38.5°C), rash, and severe arthralgia or arthritis <7 days after a mosquito bite. Clinical signs and symptoms included fever in 90% (523/584) of persons with suspected cases, headache in 65% (382/584), joint pain in 85% (497/584), and rash in 24% (141/584) (Appendix Table 1).

**Figure 1:**
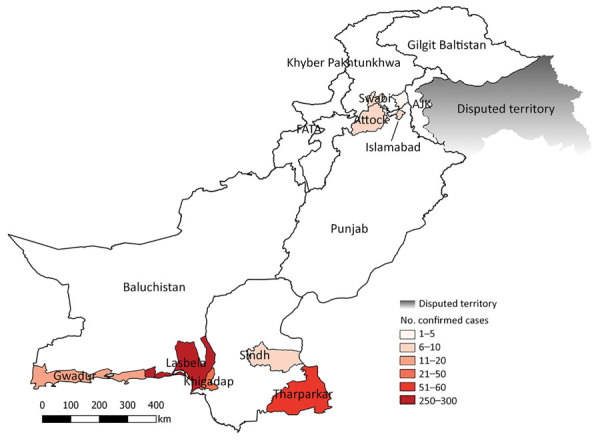
Geographic distribution of chikungunya-positive cases in Pakistan, December 20, 2016–May 31, 2017. AJK, Azad Jammu and Kashmir; FATA, Federally Administered Tribal Areas.

We used a predesigned form to collect patient demographic data, clinical information, and travel histories. We identified 495 (84.7%) suspected cases in Sindh, 52 (8.9%) in Baluchistan, 10 (1.7%) in Federal Capital Territory, 10 (1.7%) in Punjab, and 17 (2.9%) in Khyber Pakhtunkhwa (Figure 1).

We used the QIAmp Viral RNA Mini Kit (QIAGEN, https://www.qiagen.com) to extract RNA from serum samples, according to the manufacturer’s protocol. We conducted one-step real-time reverse transcription-PCR (rRT-PCR) on the ABI7500 platform (Applied Biosystems, https://www.thermofisher.com) following guidelines from the U.S. Centers for Disease Control and Prevention emergency use authorization for Trioplex Real-Time RT-PCR assay (*7*). We used CHIKV-specific oligonucleotide primers to detect and sequence the envelope 1 (E1) and nonstructural protein 1 (NSP1) genes (*8*). We conducted phylogenetic analysis of partial E1 (n = 12) and NSP1 (n = 21) by the maximum-likelihood method in MEGA version 6 (https://www.megasoftware.net). We detected CHIKV IgM by using Anti-Chikungunya Virus ELISA (Euroimmune, https://www.euroimmun.com) commercial kits.

We collected and analyzed epidemiologic data, conducted bivariate analysis, and calculated p values by using SPSS Statistics 16.0 (IBM, https://www.ibm.com). We predicted the atomic structure of the E1 protein by using the Semiliki Forest virus (PDB ID: 2XFC) as a model in UCSF Chimera version 1.11.2 (University of California, San Francisco, https://www.cgl.ucsf.edu/chimera).

We confirmed CHIKV by real-time reverse transcription PCR in 411 (70.3%) patients. The mean age of CHIKV-positive case-patients was 31.8 years (SD + 15.7 years); most (25.5%) were 21–40 years of age (Appendix Figure 1). Children ≤10 years of age had more rashes (42%) than adults, but 91% of patients 11–20 years of age and 40% of patients 21–30 years of age had joint pain and swelling. 

Of representative samples (n = 154), 85% (131/154) were positive by PCR; 67% (88/131) by IgM ELISA, of which 33% (43/131) had detectable CHIKV IgM; and 6% (8/131) of cases were confirmed by both methods. PCR was more sensitive <3 days after fever onset; IgM ELISA was more sensitive >3 days after fever onset.

**Figure 2:**
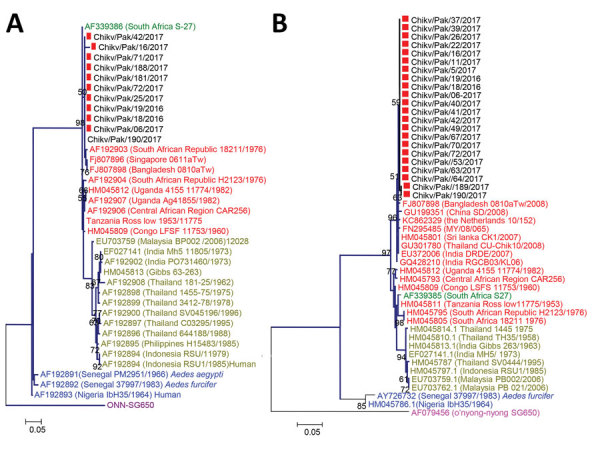
Phylogenetic tree of chikungunya viruses collected from patients in Pakistan, December 20, 2016–May 31, 2017 (red squares), and reference viruses. The tree was generated by the maximum-likelihood method based on the nucleotide sequence of the partial envelope 1 (A) and nonstructural protein 1 (B) genes. Red text indicates East/Central/South African genotype; yellow text indicates Asian genotype; green text indicates South African genotype; blue text indicates West African genotype; and purple text indicates o’nyong-nyong virus ancestral strain. GenBank accession numbers are provided for reference viruses. Scale bars indicate nucleotide substitutions per site.

Partial E1 (294 bp) and nsP1 (354 bp) gene sequences showed 99.9% similarity to strains of Indian Ocean lineage from the ECSA genotype (Figure 2). CHIKV strains in Pakistan had 99.9% homology with viruses from India, Singapore, and Bangladesh. The E1 protein of isolates from Pakistan diverged only 0.01% from prototype S-27 from Africa.

We noted substitutions in E1 genes at T98A, S111T, A145T, and K157N (Appendix Table 2) and at K128T in the NSP1 genes. We analyzed potential glycosylation sites in E1 and compared these with an o’nyong-nyong virus strain (GenBank accession no. AF079456). The E1 gene revealed a single conserved N-linked glycosylation site at N141 (Appendix Figure 2).

## Conclusions

We noted a high rate of CHIKV infection, 70% (411/584), among suspected cases; most, 37% (153/411), occurred during May, early in monsoon season, similar to a 1963 outbreak in India that coincided with monsoon season, July–December. Another study in India noted an increase in CHIKV infections during and after monsoon season, possibly reflecting the favorable breeding conditions for *Ae. albopictus* and *Ae. aegypti* mosquitoes (*9*).

With a population of >180 million, Pakistan is the world’s sixth most populous country and the second most urbanized nation in South Asia; 36% of the population resides in cities. Pakistan and other countries in Asia are experiencing harsher summers and milder winters, conditions that increase outbreaks of arboviruses likely by expanding arthropod vector breeding seasons (*10*). In addition, outbreaks can intensify in poor sanitary conditions in parts of the region. CHIKV could also be introduced into nonendemic areas by travelers with viremia, leading to local transmission (*11*). Speculated risk factors in Sindh during 2016 included population movement across the country, as well as high vector density, poor sanitation, and susceptible populations (*10*).

Genomic and serologic assays confirmed CHIKV infection 3–5 days after patients’ fever onset. We noted more CHIKV cases in persons >20 years of age. However, persons <20 years of age more frequently exhibited rashes and arthralgia, similar to results from a previous study during a 2007 outbreak in Kerala, India (*12*).

CHIKV Asian genotype circulates in Southeast Asia, where *Ae. albopictus* mosquitoes have been expanding during the previous 60 years (*13*). In CHIV-endemic settings in Asia, 2 independent E1 gene mutations in A226V and T98A could enable virus adaptation to this mosquito species. In contrast, for Indian Ocean lineage strains, the same fitness advantage and selection efficiency could be gained by the acquisition of a single T98A amino acid substitution (*13*).

Chikungunya should be considered as a diagnosis in persons who report fever, rash, or arthralgia, especially those returning from travel to virus-endemic areas (*14*). Chikungunya cases increased in Karachi and adjoining areas of Pakistan within months after massive outbreaks in India in 2016 (*15*). CHIKV in those outbreaks had high sequence homology to isolates from Pakistan, ascribing to the ECSA Indian Ocean lineage. Because no licensed CHIKV vaccine is available, public health officials should urge adoption of measures to prevent mosquito bites, such as use of repellents and mosquito nets. 

Our study had some limitations. We did not obtain information on patient outcomes or clinical management. Our results might also underreport cases because Pakistan does not have a nationwide surveillance system for CHIKV.

In summary, we report on the molecular epidemiology of CHIKV genotypes circulating in Pakistan during 2016–2017. Sustained surveillance for CHIKV is needed to monitor the extent of virus circulation in subsequent years. Identification of genotypes and monitoring for mutations that might facilitate transmission fitness for CHIKV in mosquito vectors can improve public health response. 

Appendix Additional information on chikungunya virus in Pakistan, 2016–2017.
