# Supplementary material for: Emergence of Chikungunya Virus, Pakistan, 2016–2017
Source: Emerg Infect Dis. 2020 Feb;26(2):307–10. doi: 10.3201/eid2602.171636 (PMC6986857; doi:10.3201/eid2602.171636)
Supplement: Appendix — Additional information on chikungunya virus in Pakistan, 2016–2017. [file 17-1636-Techapp-s1.pdf]

Article DOI: https://doi.org/10.3201/eid2602.171636

Emergence of Chikungunya Virus, Pakistan, 2016–2017

Appendix

Appendix Table 1. Clinical and epidemiologic characteristics of chikungunya positive and negative cases, Pakistan, 2016–2017*

Characteristics	Possible cases, no. (%) n = 584	CHIKV positive, no. (%) n = 411 (70.3)	CHIKV negative, no. (%) n = 173 (29.7)	p value
Sex				
M	277 (47.5)	192 (46.7)	85 (49.1)	0.533
F	307 (52.5)	219 (53.3)	88 (50.9)	
Age (years)				
≤10	38 (6.5)	31 (7.5)	7 (4.0)	0.62
11–20	117 (20.0)	79 (19.2)	38 (21.9)	
21–30	146 (25.5)	105 (25.5)	41 (23.7)	
31–40	122 (20.9)	89 (21.7)	33 (19.1)	
41–50	81 (13.9)	54 (13.1)	27 (15.6)	
>50	80 (13.7)	53 (12.9)	27 (15.6)	
Clinical manifestations				
Fever	523 (89.6)	368 (89.5)	155 (89.6)	0.225
Headache	382 (65.4)	282 (68.6)	100 (57.8)	0.002
Eye pain	174 (29.8)	143 (34.8)	31 (17.9)	0.001
Muscle pain	396 (67.8)	296 (72.0)	100 (57.8)	0.001
Joint pain	497 (85.1)	361 (87.8)	136 (78.6)	0.001
Swollen joints	210 (36.0)	155 (37.7)	55 (31.8)	0.11
Nausea or vomiting	253 (43.3)	190 (46.2)	63 (36.4)	0.01
Diarrhea	51 (8.7)	38 (9.2)	13 (7.5)	0.45
Chills	143 (24.5)	107 (26.0)	36 (20.8)	0.13
Cough	56 (9.5)	45 (10.9)	11 (6.4)	0.07
Abdominal pain	81 (13.9)	57 (13.9)	24 (13.9)	0.928
Fatigue	297 (50.9)	237 (57.7)	60 (34.7)	0.001
Rash	141 (24.1)	97 (23.6)	44 (25.4)	0.72

*CHIKV, chikungunya virus.

Appendix Table 2: Nucleotide and amino acid variations of envelop 1 gene in chikungunya virus from samples collected in Pakistan compared with East/Central/South African lineage and prototype S-27 from Africa

	Envelope 1 structural protein									
Nucleotide position	10254	10258	10285	10287	10299	10324	10377	10426	10464	10512
Amino acid variation				T98A		S111T		A145T	K157N	
onyong-nyong virus	C	T	G	A	A	T	A	T	G	T
S-27 strain	T	T	G	T	A	T	C	A	G	T
Ross strain	T	T	G	T	A	T	C	A	G	C
Asian	C	T	A	C	A	T	C	G	G	C
West Africa	T	T	G	C	A	T	G	G	G	T
CHIKV/Pak/06/2016	C	T	G	T	G	T	T	A	G	T
CHIKV/Pak/18/2016	C	G	G	T	G	T	T	A	G	T
CHIKV/Pak/19/2016	C	G	G	T	G	T	T	A	G	T
CHIKV/Pak/16/2017	C	T	G	T	G	A	T	A	T	T
CHIKV/Pak/25/2017	C	G	G	T	G	T	T	A	G	T
CHIKV/Pak/42/2017	C	T	G	T	G	T	T	A	G	T
CHIKV/Pak/71/2017	C	T	G	T	G	T	T	A	G	T
CHIKV/Pak/72/2017	C	T	G	T	G	T	T	A	G	T
CHIKV/Pak/181/2017	C	T	G	T	G	T	T	A	G	T
CHIKV/Pak/188/2017	C	T	G	T	G	T	T	A	G	T
CHIKV/Pak/190/2017	C	T	G	T	G	T	T	A	G	T

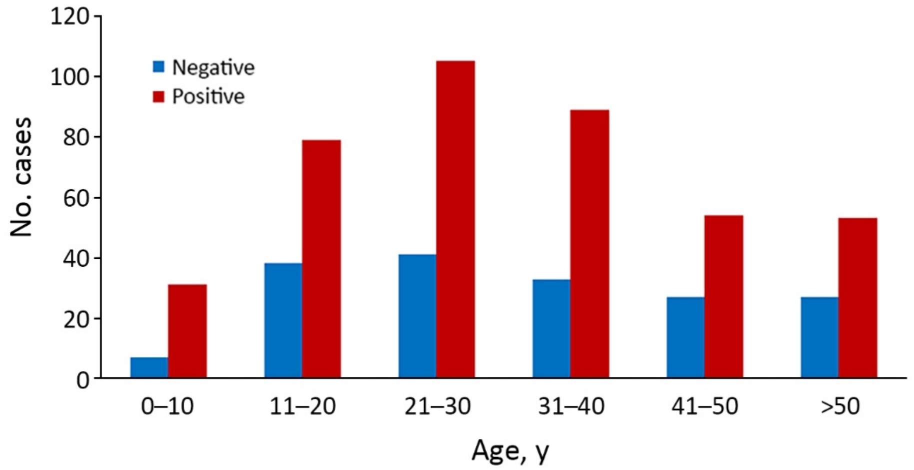

Appendix Figure 1. Distribution of chikungunya cases among age groups in Pakistan, December 20, 2016–May 31, 2017.

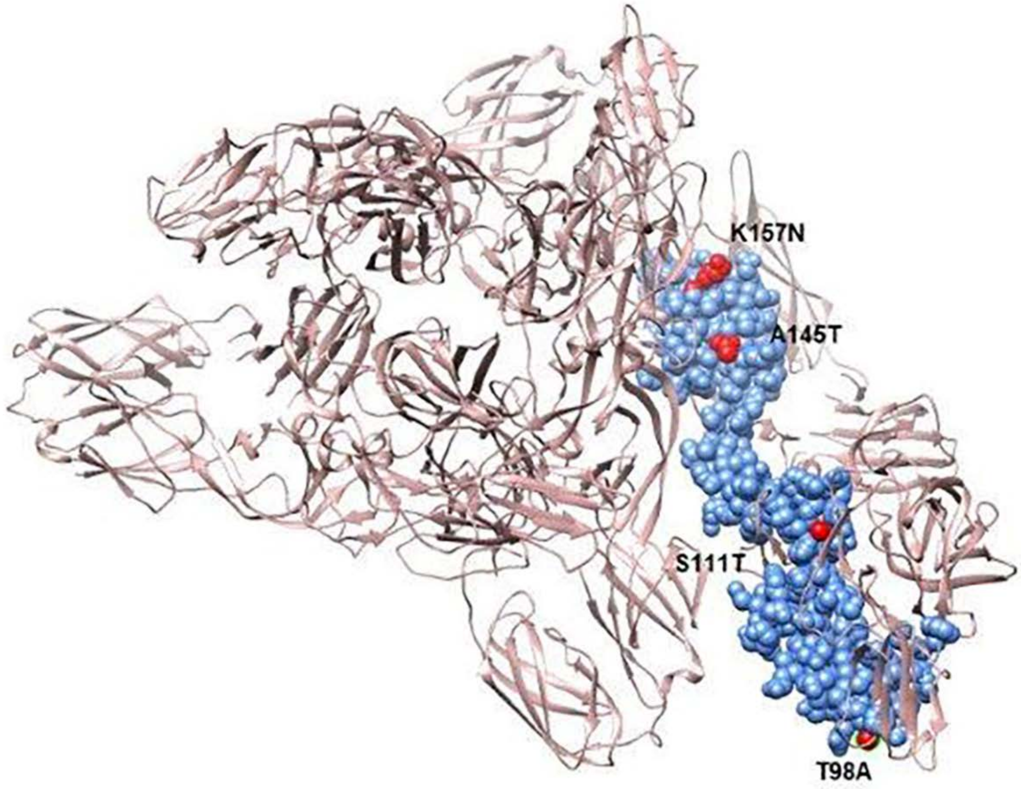

Appendix Figure 2. Predicted atomic structure of CHIKV envelop 1 (E1) protein, constructed based on the atomic structure of Semiliki Forest virus (SFV) (PDB ID: 2XFC). Molecular graphics and analyses performed with UCSF Chimera version 1.11.2 (https://www.cgl.ucsf.edu/chimera), developed by the Resource for Biocomputing, Visualization, and Informatics at the University of California, San Francisco, with support from NIH P41-GM103311.
